# Co-Exercise and Mood: The Role of Relationship Type in the Link Between Physical Activity and Positive Affect

**DOI:** 10.1093/geroni/igaf122.2080

**Published:** 2025-12-31

**Authors:** Nathan Lewis, Yoonseok Choi, Maureen Ashe, Ken Madden, Rachel Murphy, Wolfgang Linden, Denis Gerstorf, Christiane Hoppmann

**Affiliations:** The University of British Columbia, Vancouver, British Columbia, Canada; Simon Fraser University, Vancouver, British Columbia, Canada; The University of British Columbia, Vancouver, British Columbia, Canada; The University of British Columbia, Vancouver, British Columbia, Canada; The University of British Columbia, Vancouver, British Columbia, Canada; The University of British Columbia, Vancouver, British Columbia, Canada; Humboldt University Berlin, Humboldt University Berlin, Berlin, Germany; UBC, Vancouver, British Columbia, Canada

## Abstract

Physical activity is known for its positive health benefits, including improvements in mood. While research supports the association between physical activity and elevated positive affect, the possibly unique role of exercising with close social connections (i.e., co-exercise), such as spouses, friends, and family, in enhancing these mood benefits remains less understood. This study used ambulatory assessment data from 140 dyads, each consisting of an older adult (60+) and a close social connection (56.6% spouses, 30.2% friends or family members; aged 21-95 years, M = 68.59 years, SD = 10.56). Participants completed a 10-day assessment period with morning and evening surveys on affect (0-100), along with self-reported and accelerometer-measured physical activity. Multilevel models examined the relationship between physical activity and end-of-day positive affect and whether these associations varied by relationship type. A moderation analysis assessed whether the effect of co-exercise on mood differed by relationship type (spouse/partner vs. friend/family). Participants reported engaging in physical activity with their study partner on 35% of days. Higher self-reported physical activity was associated with higher positive affect (b = 0.13, SE = 0.01, p<.001), and this effect was stronger on days when participants exercised with their study partner (b = 1.44, SE = 0.69, p=.039). However, the effect of co-exercise on positive affect did not differ by relationship type. Future analyses will explore whether a similar pattern is seen with accelerometer-measured physical activity. These findings suggest that co-exercise may enhance the mood benefits of physical activity, regardless of relationship type, and may be valuable in interventions supporting the physical and mental health of older adults.

